# Clinical characteristics of bloodstream infection by *Parvimonas micra*: retrospective case series and literature review

**DOI:** 10.1186/s12879-020-05305-y

**Published:** 2020-08-05

**Authors:** Tsuyoshi Watanabe, Yuki Hara, Yusuke Yoshimi, Yoshiro Fujita, Masamichi Yokoe, Yoshinori Noguchi

**Affiliations:** 1 Department of Rheumatology, Chubu Rosai Hospital, 2-10-15, Komei-cho, Minato-ku, Nagoya, Aichi 455-8530 Japan; 2 Department of Clinical Laboratory, Japanese Red Cross Nagoya Daini Hospital, Nagoya, Aichi 466-8650 Japan; 3 Department of General Internal Medicine, Japanese Red Cross Nagoya Daini Hospital, Nagoya, Aichi 466-8650 Japan

**Keywords:** *Parvimonas micra*, Bacteremia, MALDI-TOF MS, Gram-positive anaerobic bacteria

## Abstract

**Background:**

Gram-positive anaerobic (GPA) bacteria inhabit different parts of the human body as commensals but can also cause bacteremia. In this retrospective observational study, we analyzed GPA bacteremia pathogens before (2013–2015) and after (2016–2018) the introduction of the matrix-assisted laser desorption ionization–time of flight mass spectrometry (MALDI-TOF MS).

**Method:**

We conducted a retrospective observational study by searching the microbiology database to identify all positive GPA blood cultures of patients with GPA bacteremia diagnosed using the new technique, MALDI-TOF MS, between January 1, 2016 and December 31, 2018; and using a conventional phenotypic method between January 1, 2013 and December 31, 2015 at a single tertiary center in Japan. *Parvimonas micra* (*P. micra*) (17.5%) was the second most frequently identified GPA (MALDI-TOF MS); we then retrospectively reviewed electronic medical records for 25 *P. micra* bacteremia cases at our hospital. We also conducted a literature review of published cases in PubMed from January 1, 1980, until December 31, 2019; 27 cases were retrieved.

**Results:**

Most cases of *P. micra* bacteremia were identified after 2015, both, at our institute and from the literature review. They were of mostly elderly patients and had comorbid conditions (malignancies and diabetes). In our cases, laryngeal pharynx (7/25, 28%) and gastrointestinal tract (GIT; 6/25, 24%) were identified as the most likely sources of bacteremia; however, the infection source was not identified in 9 cases (36%). *P. micra* bacteremia were frequently associated with spondylodiscitis (29.6%), oropharyngeal infection (25.9%), intra-abdominal abscess (14.8%), infective endocarditis (11.1%), septic pulmonary emboli (11.1%), and GIT infection (11.1%) in the literature review. Almost all cases were treated successfully with antibiotics and by abscess drainage. The 30-day mortalities were 4 and 3.7% for our cases and the literature cases, respectively.

**Conclusions:**

Infection sites of *P. micra* are predominantly associated with GIT, oropharyngeal, vertebral spine, intra-abdominal region, pulmonary, and heart valves. Patients with *P. micra* bacteremia could have good prognosis following appropriate treatment.

## Background

Anaerobic bacteria are major components of the microflora on the mucosal membranes in humans, and several hundred distinct species of anaerobic microorganisms have been identified by classical and molecular methods. Gram-positive anaerobes (GPAs) are found in the commensal microbiota of the digestive tract, with some species colonizing the urogenital tract and skin. GPAs include *Clostridium* spp., Gram-positive anaerobic cocci (GPAC), and Gram-positive non-spore-forming anaerobes (GPNAs) [1]. As reported, *Clostridium* spp., GPAC, and GPNAs account for 20.7, 14.7, and 6% of all anaerobic hematic isolates, respectively [1].

*Parvimonas micra* (*P. micra*) is a member of GPAC normally found in the oral cavity, respiratory system, gastrointestinal tract (GIT), and the female genitourinary tract. Originally known as *Peptostreptococcus micros*, the organism was reclassified as *P. micra* in 2006 [2]. *P. micra* is one of the bacterial species most frequently isolated from infected root canals of teeth with chronic apical periodontitis [3]. *P. micra* has also been implicated in meningitis [4], cervical and brain abscess [5, 6], infective endocarditis (IE) [7], and spondylodiscitis [8]. Recently, Badri et al. demonstrated that *P. micra* was one of the most commonly isolated GPAC species and characteristics of *Parvimonas* spp. bacteremia (mostly *P. micra*) were discussed [9]. However, due to historical difficulties with laboratory identification, there is a paucity of published data about the clinical characteristics or mortality due to bloodstream infection by *P. micra*.

A new technique, matrix-assisted laser desorption ionization–time of flight mass spectrometry (MALDI-TOF MS), was reported to correctly identify 91.2% of anaerobic isolates at the species level compared to 16S rRNA sequencing as the gold standard of diagnosis [10]. MALDI-TOF MS enables accurate and timely species classification of anaerobic organisms for appropriate treatment.

In this study, we retrospectively analyzed the causative pathogens of GPA bacteremia before (2013–2015) and after (2016–2018) the introduction of MALDI-TOF MS in a tertiary medical center. *P. micra* was found to be the second most commonly identified causative organism of GPA bacteremia using MALDI-TOF MS, therefore, we described the clinical features and laboratory diagnosis of *P. micra* bacteremia. Finally, we conducted a review of literatures on previously reported *P. micra* bacteremia cases.

## Methods

### Anaerobic bacterial isolation

Blood culture samples were incubated for 7 days in the Bact/Alert 3D instrument (bioMérieux, Marcy-l’Étoile, France) per the manufacturer’s protocol. Bact/Alert FN (anaerobic) blood culture bottles were used at the laboratory throughout 2015; Bact/Alert FN plus (anaerobic) bottles were used thereafter. Subcultures were streaked onto the Centers for Disease Control and Prevention anaerobic blood agar plates (BD Biosciences, Franklin Lakes, NJ, USA) and incubated under anaerobic conditions for 2 days. If bacterial growth was insufficient, the incubation period was extended.

### Bacterial identification

Anaerobic bacteria were identified using RapIDTM ANA II (AMCO, Inc., Tokyo, Japan) before the introduction of MALDI-TOF MS in our laboratory. Since January 2016, we have used the MALDI Biotyper (Bruker Daltonics, Inc., Billerica, MA, USA) for bacterial identification. Bacterial isolates of single colonies were identified using the plate extraction method as previously described [11]. MALDI-TOF target plates were inoculated with samples from freshly grown bacterial colonies, overlaid with 1 μL of 70% formic acid (FUJIFILM, Tokyo, Japan), and then overlaid with 1 μL matrix (α-cyano-4-hydroxycinnamic acid). The bacterial test standard (Bruker Daltonics, Inc.) was used for instrument calibration. Mass spectra were analyzed in an m/z range of 2000–20,000. MALDI Biotyper software version 3.0 and the most recent MALDI Biotyper libraries were used for bacterial identification.

For each strain of anaerobic bacteria, two preparations of colony/sample material were analyzed. The Biotyper software compares each sample’s mass spectrum to the reference mass spectrum in the database, calculates an arbitrary unit score value between 0 and 3 reflecting the similarity between the sample and reference spectrum, and displays the top 10 matching database records. Log scores were interpreted according to the manufacturer’s instructions. Log scores of > 2.0; > 1.7 but < 2.0; and < 1.7 indicate identification with high confidence, low confidence, and unreliable identification for the specie level, respectively.

### Antimicrobial susceptibility test

Antimicrobial susceptibility testing was performed for most isolates using the broth dilution method using ABCM broth (Eiken Chemicals, Tokyo, Japan). Bacteria were tested against the following antimicrobial agents: penicillin, ampicillin/sulbactam, piperacillin/tazobactam, meropenem, and clindamycin. Minimum inhibitory concentrations (MIC) were routinely reported and interpreted according to Clinical and Laboratory Standards Institute (CLSI) M100 S28 (2018) breakpoints for anaerobes.

### Case ascertainment and clinical data collection

This retrospective study was conducted at Japanese Red Cross Nagoya Daini Hospital, an 812-bed tertiary medical center in Nagoya, Japan. By searching our microbiology database, we identified all positive GPA blood cultures from January 2013 through December 2018. GPA bacteremia was identified by conventional phenotypic methods from January 2013 to December 2015 and by MALDI-TOF MS from January 2016 to December 2018. As for *P. micra*, bacteremia was expansively searched from January 2011 to December 2019, in that period, medical records were available for analyzing *P. micra* bacteremia.

Clinical data were collected from medical records. Bacteremia was considered clinically relevant when a patient had one or more positive blood cultures for GPA with clinical evidence consistent with infection: temperature > 38 °C or elevated serum concentrations of C-reactive protein (> 40 mg/L), or the source of infection was definitively identified. Community-onset bacteremia was defined as infection acquired outside of the hospital setting, with a positive blood culture obtained within 48 h of admission. Nosocomial bacteremia was defined as infection acquired while being administered treatment within a healthcare setting, with a positive blood culture obtained more than 48 h after admission. Charlson Comorbidity Score was used to assess comorbidities [12]. Illness severity was assessed using the Pitt bacteremia score [13].

### Literature review

We searched the PubMed databases for journal articles, using the search terms “*Parvimonas micra*” or “*Peptostreptococcus micros*” and “bacteremia” or “Infection.” Articles published from January 1, 1980 to December 31, 2019 were included. Articles were individually assessed for a case of *P. micra* bacteremia based on the title and/or abstract and/or text*.* All articles were reviewed to ensure no redundancy of cases, and that adequate information was available for inclusion in the review.

## Results

In our analysis, 70 clinically relevant cases of GPA-associated bacteremia were detected in 2013–2015, whereas 126 were detected in 2016–2018 after introducing MALDI-TOF MS (sFigure 1). In 2016–2018, *Clostridium perfringens* was the most frequently identified causative organism (23/126 cases, 18.3%) of GPA bacteremia, followed by *P. micra* (22/126 cases, 17.5%), and *Eggerthella lenta* (12/126 cases, 9.5%) (Table 1). In 2016–2018, more cases of GPA bacteremia were identified at the specie level (83.3%, 105/126 cases) compared to those identified in 2013–2015 (62.9%, 44/70 cases). *P. micra* bacteremia was observed in 23 cases using either MALDI-TOF MS or conventional phenotypic method in our institute.

**Table 1:** Lists of Gram-positive anaerobes (GPA) s identified in blood cultures using MALDI-TOF MS (2016–2018) and conventional phenotypic method (2013–2015)

2016–2018	Number (%)	2013–2015	Number (%)
Identified GPA at species level		Identified GPA at species level	
*Clostridium perfringens*	23 (18.3)	*Clostridium perfringens*	26 (37.1)
*Parvimonas micra*	22 (17.5)	*Anaerococcus prevotii*	6 (8.6)
*Eggerthella lenta*	12 (9.5)	*Clostridium innocuum*	2 (2.9)
*Clostridium ramosum*	5 (4.0)	*Eubacterium limosum*	2 (2.9)
*Bifidobacterium breve*	5 (4.0)	*Anaerococcus tetradius*	1 (1.4)
*Actinomyces neuii*	4 (3.2)	*Clostridium butyricum*	1 (1.4)
*Lactobacillus rhamnosus*	3 (2.4)	*Clostridium septicum*	1 (1.4)
*Actinomyces oris*	2 (1.6)	*Clostridium sporogenes*	1 (1.4)
*Brevibacterium ravenspurgense*	2 (1.6)	*Clostridium subterminale*	1 (1.4)
*Clostridium clostridioforme*	2 (1.6)	*Eggerthella lenta*	1 (1.4)
*Eubacterium limosum*	2 (1.6)	*Paraclostridium bifermentans*	1 (1.4)
*Lactobacillus fermentum*	2 (1.6)	*Parvimonas micra*	1 (1.4)
*Lactobacillus paracasei*	2 (1.6)		
*Actinomyces meyeri*	1 (0.7)		
*Actinomyces odontolyticus*	1 (0.7)		
*Actinomyces turicensis*	1 (0.7)		
*Actinotignum schaalii*	1 (0.7)		
*Bifidobacterium adolescentis*	1 (0.7)		
*Bifidobacterium dentium*	1 (0.7)		
*Bifidobacterium longum*	1 (0.7)		
*Brevibacillus brevis*	1 (0.7)		
*Clostridioides difficile*	1 (0.7)		
*Clostridium bifermentans*	1 (0.7)		
*Clostridium butyricum*	1 (0.7)		
*Clostridium innocuum*	1 (0.7)		
*Clostridium paraputrificum*	1 (0.7)		
*Clostridium septicum*	1 (0.7)		
*Clostridium symbiosum*	1 (0.7)		
*Hungatella hathewayi*	1 (0.7)		
*Lactobacillus gasseri*	1 (0.7)		
*Lactobacillus salivarius*	1 (0.7)		
*Paraclostridium bifermentans*	1 (0.7)		
Unidentified GPA at species level		Unidentified GPA at species level	
Undetermined anaerobic gram positive cocci	18 (14.3)	Undetermined anaerobic gram positive cocci	14 (20.0)
*Clostridium* species	2 (1.6)	Undetermined anaerobic gram positive rod	3 (4.3)
Undetermined anaerobic gram positive rod	1 (0.7)	*Propionibacterium* species	3 (4.3)
		*Clostridium* species	3 (4.3)
		*Peptostreptococcus* species	2 (2.9)
		*Lactobacillus* species	1 (1.4)
Total	126	Total	70

In addition to these 23 cases, we identified 2 cases of *P. micra* bacteremia in 2011 and 2019, where medical records data were available and sufficient to analyze the clinical characteristics. Details of a total of 25 cases with bacteremia caused by *P. micra* that we identified are shown in Table 2. The median age of the 25 patients with *P. micra* bacteremia was 83.0 years (range, 58–92 years), and 14/25 (56%) patients were male. Most patients (23/25, 92%) had community-onset disease. Nearly all patients (23/25, 92%) presented with fever; however, focal symptoms such as sore throat (1/25, 4%) and abdominal pain (1/25, 4%) were less frequently observed. The most common comorbidities were cancer (9/25, 36%) and diabetes (6/25, 24%). The laryngeal pharynx (7/25, 28%) and GIT (6/25, 24%) were identified as the most likely sources of bacteremia. The infection source was not identified in 9 cases (9/25, 36%). Twelve patients (48%) had polymicrobial bacteremia. The various pathogens associated with concomitant polymicrobial infection included *Solobacterium moorei*, *Staphylococcus capitis*, *Fusobacterium nucleatum*, *Pseudomonas citronellolis*, *Bacillus subtilis*, *Streptococcus oralis*, *Streptococcus constellatus*, *Staphylococcus lugdunensis*, *Veillonella parvula*, *Actinomyces meyeri*, *Prevotella intermedia*, *Atopobium parvulum*, *Streptococcus anginosus, and Prevotella oris, Corynebacterium spp*. Most *P. micra* isolates were susceptible to penicillin, ampicillin-sulbactam, piperacillin-tazobactam, and meropenem; however, clindamycin showed slightly elevated MIC (susceptibility rate, 86.7%) (Table 2). Most cases (24/25, 96%) were treated successfully, and surgery was performed in one patient with gastroesophageal junction carcinoma who had infection due to postoperative anastomotic leakage.

**Table 2:** Clinical characteristics of *P. micra* in case series, and in the literature cases

Characteristic		Patients [no. (%) or as indicated] with:	
		Our case series (25)	Literature cases (27)
Age	yr: median (range)	83.0 (58–92)	59.0 (23–94)
Gender	No.male/no.famale (ratio)	14/11 (1.3)	15/12 (1.3)
Community onset/nosocomial		23/2	26/1
Method of pathogen detection	MALDI-TOF MS	23	6
	16S rRNA sequencing	0	3
	Biochemical test	2	4
	Gas chromatography	0	6
Symptom	Fever, no. (%)	23 (92.0)	12 (44.4)
	Headache, no. (%)	0	2 (7.4)
	Pharyngeal symptoms, no. (%)	1 (4.0)	0
	Shortness of breath, no. (%)	0	2 (7.4)
	Abdominal symptoms, no. (%)	1 (4.0)	3 (11.1)
	Vomiting, no. (%)	1 (4.0)	1 (3.7)
	Diarrhea, no. (%)	1 (4.0)	0
	Myalgia, no. (%)	0	4 (14.8)
	Shaking and/or chill, no. (%)	9 (36.0)	4 (14.8)
	Low back pain, no. (%)	0	6 (22.2)
Underlying health status	Steroid usage, no. (%)	1 (4.0)	0
	Diabetes, no. (%)	6 (24.0)	3 (11.1)
	Heart valve replacement, no. (%)	0	2 (7.4)
	Malignancies, no. (%)	9 (36.0)	3 (11.1)
	Diverticular disease, no. (%)	1 (4.0)	1 (3.7)
	Abdominal operation within 4 weeks, no. (%)	1 (4.0)	0
	Gastrointestinal perforation, no. (%)	1 (4.0)	0
	Gynecologic disease, no. (%)	0	3 (11.1)
	Charlson comorbidity score, median (range)	5 (0–7)	N/R^a^
Focus or likely source of infection	Meningitis, no. (%)	0	1 (3.7)
	Oropharyngeal, no. (%)	7 (28.0)	7 (25.9)
	Endocarditis, no. (%)	0	3 (11.1)
	Lung, no. (%)	1 (4.0)	0
	Septic pulmonary emboli, no. (%)	1 (4.0)	3 (11.1)
	Hepatobiliary (liver, pancreas, gallbladder, biliary), no. (%)	1 (4.0)	1 (3.7)
	Gastrointestinal tract, no. (%)	6 (24.0)	3 (11.1)
	Intraabdominal abscess, no. (%)	0	4 (14.8)
	Pylephlebitis, no. (%)	0	2 (7.4)
	Spondylodiscitis, no. (%)	1 (4.0)	8 (29.6)
	Bacteremia with unknown source, no. (%)	9 (36.0)	4 (14.8)
Polymicrobial bacteremia	no. (%)	12/25 (48)	5/27 (18.5)
Pitt bacteremia score	median (range)	1 (0–13)	N/R
Antibiotic sensitivity testing ^b^	Penicillin, no. (%)	22/22 (100) ^b^	10/10 (100)
	Ampicillin-sulbactam, no. (%)	12/12 (100) ^b^	4/4 (100)
	Piperacillin-tazobactam, no. (%)	12/12 (100) ^b^	5/5 (100)
	Meropenem, no. (%)	19/19 (100) ^b^	5/5 (100)
	Clindamycin, no. (%)	13/15 (86.7) ^b^	9/9 (100)
All-cause mortality at:	30 days, no. (%)	1/25 (4.0)	1/27 (3.7)

^a^*N/R* not reported

^b^Percentage of susceptible isolates based on CLSI M100 S28 (2018) in our case series, presented as number of isolates with susceptibility/ number of isolates measured (susceptibility rate %)

Literature review identified 279 articles, and these were assessed for inclusion. Overall, 251 articles were excluded based on the title and/or abstract and/or text or due to duplication. One article was excluded due to duplication with our case series. There were 27 reported cases of *P. micra* bacteremia (sTable 1, 26 cases in English and 1 case in Japanese). We also found that Badri et al. reported 96 cases of *P. micra* bacteremia. However, since combined data of clinical characteristics about *P. micra* and other *Parvimonas* spp. were demonstrated in that study, we described the results of the literature by Badri et al. in the section of discussion [9].

In 27 literature cases, *P. micra* bacteremia was frequently associated with spondylodiscitis (8/27, 29.6%), oropharyngeal infection (7/27, 25.9%) intraabdominal abscess (4/27, 14.8%), GIT infection (3/27, 11.1%), IE (3/27, 11.1%), and septic pulmonary emboli (3/27, 11.1%). Five patients (18.5%) had polymicrobial bacteremia, and pathogens associated with concomitant polymicrobial infection included *Gemella morbillorum*, *Atopobium rimae*, *Fusobacterium nucleatum*, *Prevotella bivia*, and *Bacteroides fragilis*. In the literature cases, *P. micra* bacteremia also had a favorable outcome (mortality, 1/27, 3.7%).

## Discussion

**Fig. 1:**
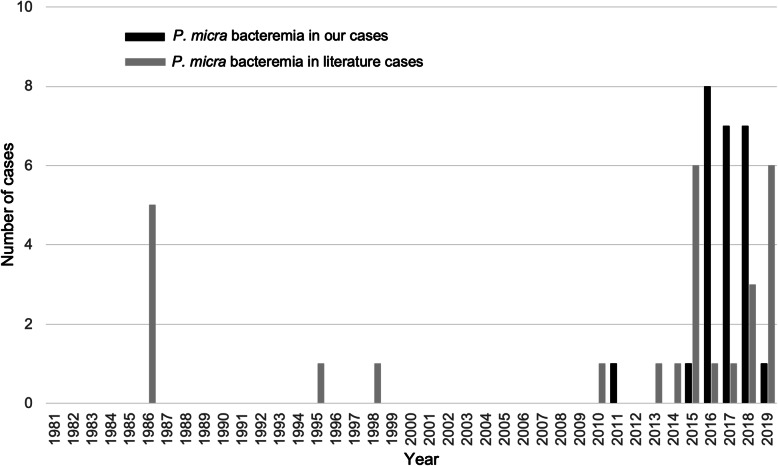
Confirmed cases of *P. micra* bacteremia during 2010–2019 in our institute, and 1980–2019 in literature review, respectively

In our cases, using MALDI-TOF MS, *P. micra* was a frequently identified causative organism of GPA bacteremia. However, *P. micra* have rarely been identified as causative pathogens of GPA bacteremia using conventional methods due to difficulties with the laboratory identification. In both ours and the literature cases, *P. micra* bacteremia cases were increasingly diagnosed after 2015 (Fig. 1) and were mostly detected using MALDI-TOF MS or 16S rRNA gene sequencing (Table 2 and sTable 1**)**. In a recent population-based study, GPAC bacteremia, identified using MALDI-TOF MS or 16S rRNA gene sequencing, was reported to be much more common than previously reported with an annual incidence of 3.4 cases per 100,000 persons per year [9], while *P. micra* was the most commonly isolated in the study. Thus, these new techniques might enable accurate diagnosis of *P. micra* bacteremia for appropriate treatment.

We reviewed 25 patients with *P. micra* bacteremia using MALDI-TOF MS or conventional phenotypic method from 2010 to 2019. We found that oropharyngeal infections (7/25, 28.0%) and GIT (6/25, 24.0%) were the primary sources of *P. micra* bacteremia; however, the sources of bacteremia in the remaining 9 patients (36.0%) were not recorded. As the presenting symptoms in these patients were non-specific (e.g., fever, shake, and chills), thorough searches for the primary source of bacteremia may not have been carefully conducted in these 9 cases. In 27 literature cases, *P. micra* bacteremia were also frequently associated with oropharyngeal infection (7/27, 25.9%) and GIT infection (3/27, 11.1%) [14–31]. Other common infectious diseases of *P. micra* in the literature cases were spondylodiscitis (8/27, 29.6%), intra-abdominal abscess (4/27, 14.8%), IE (3.27, 11.1%), and septic pulmonary emboli (3/27, 11.1%) which is inconsistent with our results. Spondylodiscitis caused by *P. micra* is rare but an important infectious disease, which is sometimes diagnosed from samples of the bone or vertebral disk without positive blood culture [23]. IE due to anaerobic bacteremia account for 2–16% of all cases of IE over the past few decades [32], and *P. micra* might be one of the important anaerobic pathogens causing IE. Recently, Badri and coworkers reported clinical features about 100 cases of *Parvimonas* spp. (96 *P. micra* and 4 *Parvimonas* spp.), in which common focal infections were in the abdominal tract, bones and joints, the respiratory tract, and in skin/soft tissue, however, oropharyngeal infection were rarely identified [9]. Our case series and literature review indicated that it is necessary to note the GIT, oropharyngeal tract, pulmonary, vertebral spine, intra-abdominal region, and heart valves as infectious sites of *P. micra* bacteremia.

In our cases, 48% (12/25) of *P. micra* bacteremia had polymicrobial bacteremia. In the literature cases, 5 patients (18.5%) had polymicrobial bacteremia. Most concomitant pathogens were aerobic and anaerobic members of the oral and gastrointestinal microbiota. Badri et al. showed that 50% of *Parvimonas* spp. associated bacteremic infections were polymicrobial [9], which was equivalent to our cases. Although there was no difference in the mortality rate between patients with monomicrobial and polymicrobial anaerobic bacteremia [33], it was reported that inappropriate antibiotic selection without attention to the results of anaerobic cultures have serious consequences for patients [34]. Therefore, we should choose the antibiotics targeting identified anaerobic organisms including *P. micra*.

Most cases of *P. micra* bacteremia in our case series were successfully treated with antibiotics (mortality, 1/25, 4.0%) (Table 2). Median of Pitt bacteremia score was 1, and two of 25 cases were treated in the intensive care unit. In the literature cases, *P. micra* bacteremia also had a favorable outcome (mortality, 1/27, 3.7%). In one of the previous cases, a patient died as a consequence of the primary tumor after the completion of antibiotic therapy [21]. In a previous study, in-hospital mortality of GPAC bacteremia including *Parvimonas* spp. was 10% [9]. In contrast, the overall mortality was as high as 25% in all patients with anaerobic bacteremia [34]. Although *P. micra* can cause complicated infectious diseases including IE and spondylodiscitis, these results suggest that patients with *P. micra* bacteremia might have good prognosis following appropriate treatment.

There were some limitations to this study. First, we may have missed some cases at our institute as well as previously reported cases due to the retrospective design. Second, the cases in our case series were diagnosed in a single tertiary medical center in Japan; therefore, regional and institutional differences were not considered. Third, different blood culture bottles were used in the two study periods. Although a prior study found no difference in the detection rate of anaerobic bacteremia using Bact/Alert FN bottles or Bact/Alert FN plus bottles [35], we cannot rule out the potential impact of our transition from Bact/Alert FN to Bact/Alert FA plus bottles on identification. Lastly, the literature review on *P. micra* bacteremia was dependent on publication biases, making it impossible to address the real prevalence and incidence of *P. micra* bacteremia in clinical setting.

## Conclusion

We reported the causative pathogens of GPAs bacteremia identified by MALDI-TOF MS, and findings showed *P. micra* as the second most frequently identified GPA. Our case series and literature review showed that *P. micra* have been mainly identified in blood culture using MALDI-TOF MS and 16 s rRNA sequencing. Infection sites of *P. micra* were predominantly associated with GIT, oropharyngeal, vertebral spine, intra-abdominal region, pulmonary, and heart valves. Patients with *P. micra* bacteremia could have good prognosis following appropriate treatment. This study helps improve our understanding of the clinical characteristics of bloodstream infections by *P. micra*.

## Supplementary information

**Additional file 1: Supplemental Figure 1.** Numbers of GPAs identified or not identified at the species level from 2013 to 2018. Anaerobic bacteria were identified by a conventional phenotypic method from 2013 to 2015, and MALDI-TOF MS from 2016 to 2018.

**Additional file 2: Supplemental Table 1.**

## Data Availability

The datasets used and/or analyzed during the current study are available from the corresponding author on reasonable request.

## Abbreviations

CLSI: Clinical and Laboratory Standards Institute
GIT: Gastrointestinal tract;
GPA: Gram-positive anaerobe
GPAC: Gram-positive anaerobic cocci
GPNA: Gram-positive non-spore-forming anaerobe
IE: Infective endocarditis
MALDI-TOF MS: Matrix-assisted laser desorption ionization–time of flight mass spectrometry
MIC: Minimum inhibitory concentration
*P. micra*: *Parvimonas micra*
