# Supplementary figures and images for: Clinical characteristics of bloodstream infection by Parvimonas micra: retrospective case series and literature review
Source: BMC Infect Dis. 2020 Aug 5;20:578. doi: 10.1186/s12879-020-05305-y (PMC7405351; doi:10.1186/s12879-020-05305-y)

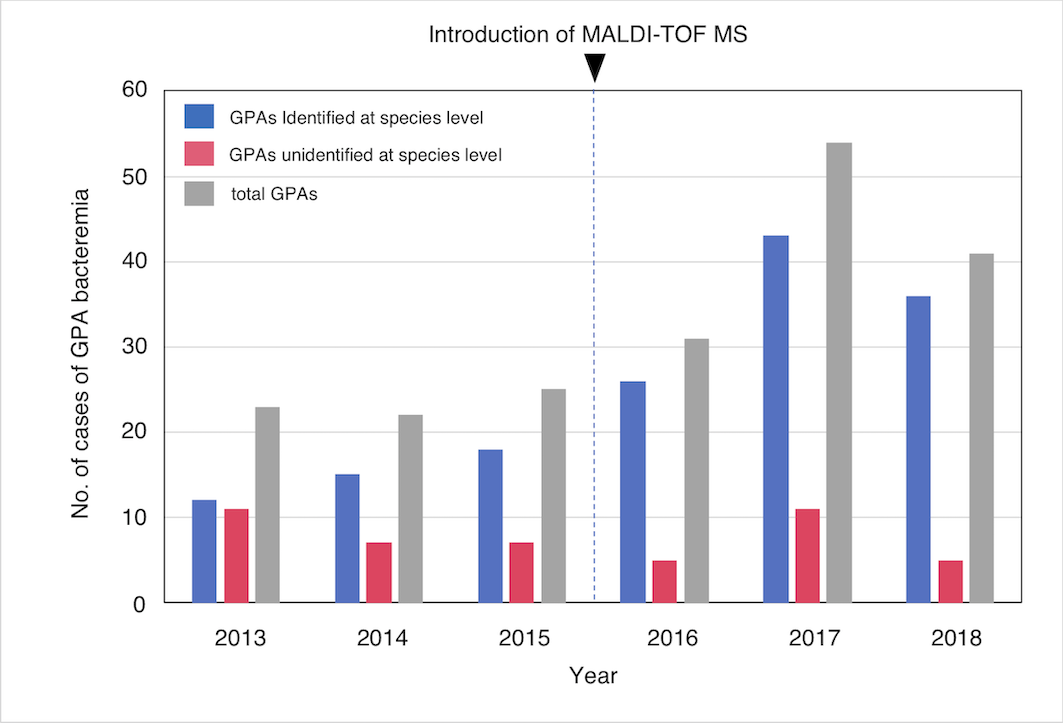

Supplement: Supplementary file 1 — Additional file 1: Supplemental Figure 1. Numbers of GPAs identified or not identified at the species level from 2013 to 2018. Anaerobic bacteria were identified by a conventional phenotypic method from 2013 to 2015, and MALDI-TOF MS from 2016 to 2018. [file 12879_2020_5305_MOESM1_ESM.tiff]
