# Supplementary material for: Clinical characteristics of bloodstream infection by Parvimonas micra: retrospective case series and literature review
Source: BMC Infect Dis. 2020 Aug 5;20:578. doi: 10.1186/s12879-020-05305-y (PMC7405351; doi:10.1186/s12879-020-05305-y)
Supplement: Supplementary file 2 — Additional file 2: Supplemental Table 1. [file 12879_2020_5305_MOESM2_ESM.docx]

**Supplemental table 1.**

| **No.** | **References** | **Age (years)/ sex** | **Clinical features** | **Infectious site** | **Possible source  of infection** | **Past medical history** | **Method of  pathogen detection** | **Coinfectious pathogen** | **Antibiotic** | **Outcome** |
| --- | --- | --- | --- | --- | --- | --- | --- | --- | --- | --- |
| 1 | Khan MS et al. 2019 [14] | 94/M | Fever, constipation, weakness | Bacteremia | colonic carcinoma | Colonic carcinoma,  diabetes mellitus, dyslipidemia | N/R | *Gamella morbillorum* (blood) | Ampicillin-sulbactam IV  2 weeks or more | Cure |
| 2 | Yoo LJH et al. 2019 [15] | 77/F | Low back pain | Spondylodiscitis, psoas abscess | N/R | Cerebrovascular accident,  hypertension, hyperlipidemia,  osteoporosis | N/R | none | Ceftriaxone IV and  oral metronidazole 8 weeks | Cure |
| 3 | Hale GR et al. 2019 [16] | 77/M | Fever, rigor, abdominal pain,  shoulder pain, shortness of breath,  weight loss | Pylepylebitis, splenic abscess | N/R | Jejunal perforation | MALDI-TOF MS | coagulase-negative Staphylococcus,  Actinomyces, Bacillus (abscess) | Ampicillin-sulbactam IV  6 weeks | Cure |
| 4 | Yun SS et al. 2019 [17] | 49/M | Shortness of breath | Lung abscess | preodontal disease | Hypertension | N/R | *Actinomyces odontolyticus* (abscess) | Ceftriaxone and clindamycin IV 26 days  and oral amoxicillin-clavulanate 6 months | Cure |
| 5 | Kim EY et al. 2019 [18] | 65/F | Altered mentality,  poor oral intake,  nausea, weakness | Liver abscess, brain abscess,  septic pulmonary emboli | N/R | none | VITEK2 16s rRNA | none | Cefotaxime and metronidazole | N/R |
| 6 | Sawai T et al. 2019 [19] | 81/M | Fever | Iliopsoas abscess | N/R | Chronic heart failure,  chronic renal failure | API ZYM | none | Ampicillin-sulbactam  IV 5 weeks | Cure |
| 7 | Boattini M et al. 2018 [20] | 85/M | Fever, shivering | Bacteremia | MRCP procedure | Choledocholithiasis, hypertention | MALDI-TOF MS | none | Penicillin G IV 2 weeks | Cure |
| 8 | Cobo F et al. 2018 [21] | 43/F | Fever, nausea, vomiting,  abdominal pain | Bacteremia | necrotic tumoral mass | Retroperitoneal leiomiosarcoma | MALDI-TOF MS | *Atopobium rimae* (blood) | Piperacillin-tazobactam and  metronidazole IV 10 days | dead |
| 9 | Ho M et al. 2018 [22] | 42/M | Fever | Endocarditis | tooth extraction | Diabetes mellitus,  mechanical mitral valve replacement  due to endocarditis | N/R | none | Penicillin G IV  (at least 6 weeks) | Cure |
| 10 | Higashi Y et al. 2017 [23] | 67/M | Low back pain | Spondylodiscitis | periodontitis | Diabetes mellitus | MALDI-TOF MS | none | Ampicillin-sulbactam IV  19 days and ampicillin IV 53 days | Cure |
| 11 | Garcia Carretero R et al. 2016 [24] | 53/M | Malaise, fever, chill,  anorexia, asthemia,  weight loss | Bacteremia | esophageal carcinoma  or periodontal disease | Alcoholic liver disease | N/R | none | Meropenem IV 2 weeks | dead |
| 12 | Gahier M et al. 2015 [25] | 59/F | Fever, cervical pain, asthenia | Spondylodiscitis | dental apical granuloma | N/R | N/R | none | Amoxicillin 14 weeks | Cure |
| 13 | Gahier M et al. 2015 [25] | 82/F | Low back pain | Spondylodiscitis | dental apical granuloma | N/R | N/R | none | Amoxicillin 6 weeks | Cure |
| 14 | Gahier M et al. 2015 [25] | 60/F | Low back pain | Spondylodiscitis | N/R | N/R | N/R | none | Amoxicillin 12 weeks | Cure |
| 15 | Ko JH et al. 2015 [4] | 61/M | Fever, headache | Meningitis | periodontitis | Chronic hepatitis B, dyslipidemia | VITEK2, 16S rRNA | none | Ceftriaxone and vancomycin IV 9days and oral metronidazole 12 days | Cure |
| 16 | Medina F et al. 2015 [26] | 23/F | Fever, headache,  paravertebral myalgia | Vertebral vein thrombosis,  spondylodiscitis, septic pulmonary emboli, retropharyngeal abscess | N/R | none | MALDI-TOF MS | none | Amoxicillin-clavulanate 10 days  and oral rifampicin 6 weeks combined with clindamycin 4 weeks | Cure |
| 17 | Pilmis B et al. 2015 [27] | 83/M | Low back pain | Spondylodiscitis, paraspinal abscess,  psoas abscess | N/R | Left hip/right knee joint surgery,  ischemic heart disease | MALDI-TOF MS | none | Amoxicillin and gentamycin IV followed by  oral clindamycin and rifampicin (a total of 3 months) | Cure |
| 18 | Uemura H et al. 2014 [8] | 85/F | Malaise, anorexia | Spondylodiscitis, paravertebral abscess | periodontitis | Hypertension | Rap ID 32A | *Fusobacterium nucleatus* (blood) | Ampicillin IV 4 weeks and  oral amoxicillin 8 weeks | Cure |
| 19 | Ubukata S et al. 2013 [28] | 46/M | Anorexia, dizziness | Orbital abscess, neck abscess,  mastoiditis, septic pulmonary emboli | N/R | N/R | 16S rRNA | none | Meropenem IV 1 week and  ampicillin IV 4 weeks | Cure |
| 20 | Minces LR et al. 2010 [29] | 63/F | Fall | Endocarditis | N/R | Bioprosthetic aortic valve and  mehcanical mitral valve replacement,  endocarditis | N/R | none | Penicillin G IV 6 weeks and  gentamycin IV for the first 2 weeks | Cure |
| 21 | Shiota T et al. 1998 [30] | 53/M | N/R | Pleuritis | N/R | Advances rectal adenocarcinoma | N/R | *Trichomonas tenax* (pleural fluid) | Ceftazidime 11 days and  clindamycin 10 days | Cure |
| 22 | Wenisch C et al. 1995 [7] | 30/F | Chills, fever,  lower abdominal pain,  malaise, weight loss | Endocarditis | N/R | Congenital subvalvular stnosis | Gas chromatography | none | Teicoplanin IV 23 days and  oral fusidic acid 2 weeks | Cure |
| 23 | Topiel MS et al. 1986 [31] | 28/F | N/R | Endometritis | N/R | C-section | Minitek system and  gas-liquid chromatography | *Prevotella bivia* (blood) | N/R | Cure |
| 24 | Topiel MS et al. 1986 [31] | 32/F | N/R | Endometritis | N/R | C-section | Minitek system and  gas-liquid chromatography | none | N/R | Cure |
| 25 | Topiel MS et al. 1986 [31] | 34/F | N/R | Endometritis | N/R | C-section | Minitek system and  gas-liquid chromatography | none | N/R | Cure |
| 26 | Topiel MS et al. 1986 [31] | 50/M | N/R | Pylephlebitis | N/R | Diverticulosis | Minitek system and  gas-liquid chromatography | *Bacteroides fragilis* (blood) | N/R | Cure |
| 27 | Topiel MS et al. 1986 [31] | 55/M | N/R | Cholangitis | N/R | Lymphoma | Minitek system and  gas-liquid chromatography | none | N/R | Cure |
|  |  |  |  |  |  |  |  |  |  |  |

N/R. not reported, IV intravenous.
